# Out-Patients and an Extension of the Almoner System

**Published:** 1913-01-25

**Authors:** 


### OUT-PATIENTS AND AN EXTENSION OF THE ALMONER SYSTEM.

BY AN ASSISTANT SECRETARY.

Every improvement in the organisation or methods of a voluntary hospital is primarily a question of finance: there is probably not an institution in Great Britain in which changes for the better could not be made if money were forthcoming to meet the expenditure entailed. In considering the advisability of an extension of the almoner system to cover both casualty and out-patients on the lines suggested by the committee appointed by the Council of King Edward's Hospital Fund for London, the first question that suggests itself is the cost. This has been estimated at 6d. per patient; that is, an addition of nearly 25 per cent. to the cost of the whole treatment now afforded to an out-patient. Such estimates must be largely a matter of guesswork; but considering that in many hospitals the organisation outlined by the committee would require practically the creation of a new department, the figure does not seem to be excessive.

Apart from the difficulty of increasing income to meet new permanent expenditure, if this sum could be devoted to the actual care of patients, methods of treatment, drugs, and surgical appliances, which are now considered to be too expensive, could be employed with very great advantage.

The committee believe that the extension of the almoner system would result in a corresponding reduction of patients; but this is hardly borne out by the suggestion that it would lead to an increase in the numbers of those sent for consultation and advice—the most expensive class. It may fairly be assumed, therefore, that the proposed change would increase the cost of the out-patient department by at least 25 per cent., and that this sum could be advantageously spent in other directions.

### The Question of Overcrowding.

The advantages claimed for an elaborate almoner system are many, and the charges made against the existing out-patient departments are weighty. Logically, both may be justified; but it is the British genius to produce from an illogical system, from a mass of half-contradictory principles and regulations, a good sound workable system which is the satisfactory result of gradual evolution rather than sudden revolution. It must remain a matter of opinion to some extent whether out-patient departments in general are overcrowded: there are certainly long hours of waiting, but this could be remedied by other means than a problematical reduction in numbers. It is equally doubtful whether general practitioners really do suffer from the existence of a hospital. If it robs them of a possible few paying patients it relieves them of an immense number of cases which cannot pay, and of some lengthy or difficult club cases.

It has been assumed all along in this matter that out-patient departments are absolutely free, and the charge of pauperisation has been logically pressed. But as a matter of fact, in different ways and to a varying extent some return is obtained in practically all hospitals from the out-patients. In my own hospital the contents of the surgery and dispensary boxes for the year 1911 worked out at very nearly 1½d. per patient. But beyond this a number of those who use the hospital are already paying towards it in one way or another, either by collections in their respective works, or by contributions to local charity sports, or by weekly payments to the Hospital Saturday Fund.

Logically, it is easy to speak of a line of destitution and to send to the Poor-Law dispensary those who fall below it. It is, however, now recognised, I believe, by the Local Government Board that no such line is capable of definition. It must also be remembered that most hospitals were endowed and are maintained for giving the best possible treatment in illness to the poor, and that it is hardly consonant with their objects to send away persons on the ground of excessive poverty to an agency whose efficiency is certainly not equal at present to that of the out-patient department of a hospital. It would seem, therefore, that in the future, as in the past, only those could be referred to the Poor-Law who are in need of food rather than medicine.

### Three Criticisms of the New System.

The criticism directed against out-patient departments appears to be theoretical rather than practical, and, though there are many shortcomings and disadvantages, they are incidental rather than inherent in the system.

If the criticism directed against existing conditions has been too severe it follows that the advantages of the elaborate system proposed have been somewhat exaggerated. That there are great advantages may fairly be admitted, but I do not propose to discuss them here for the reason that I believe the new system to be unworkable.

1. It involves a multiplication of provident dispensaries which are seldom self-supporting in the very districts where the existing hospital can hardly pay its way.

2. It depends upon a degree of labour on the part of the honorary medical staff in maintaining constant touch with general practitioners and external agencies which the staff could not undertake even if they were willing.

3. It would interfere to an impossible extent with medical education and with the rights and responsibilities of hospital medical officers.

I have put forward the foregoing tentative considerations in the hope that they may lead to criticism and comment by other hospital officers; but there remains for the charitable public the question of how far what may be called the principles of charity organisation are to be carried. Their effect has up to now been felt with benefit in almost every department of social work, but it is possible to spend too much time, energy, and money in defeating the undeserving; while as regards co-ordination the hospital patient may have more common sense than is believed in knowing what he wants and where to go for it.

The Views of the Patients.

It is worthy of note that in conducting an inquiry of this kind not much attempt has been made to obtain from the patients themselves their views of the work of out-patient departments, and I would venture to suggest in conclusion that a most fruitful field of inquiry is here open to the investigator. In every department of social reform there is a tendency to start out with preconceived theories of what the poor should need. In the case of hospitals which depend to a considerable extent for existence on the working-classes it would be specially interesting to know what the poor do need.

